# Effects of Different Surface Treatments and Accelerated Aging on Dental Zirconia—An In Vitro Study

**DOI:** 10.3390/jfb16070263

**Published:** 2025-07-16

**Authors:** Mihaela Pantea, Lucian Toma Ciocan, Vlad Gabriel Vasilescu, Georgeta Voicu, Adrian-Ionut Nicoară, Florin Miculescu, Robert Ciocoiu, Ana Maria Cristina Țâncu, Elena Georgiana Banu, Marina Imre

**Affiliations:** 1 Department of Prosthodontics, Faculty of Dentistry, “Carol Davila” University of Medicine and Pharmacy, 37 Dionisie Lupu Street, District 2, 020021 Bucharest, Romania; mihaela.pantea@umfcd.ro (M.P.); anamaria.tancu@umfcd.ro (A.M.C.Ț.); georgiana.banu@stud.umfcd.ro (E.G.B.); marina.imre@umfcd.ro (M.I.); 2 Department of Dental Prostheses Technology, Faculty of Dentistry, “Carol Davila” University of Medicine and Pharmacy, 37 Dionisie Lupu Street, District 2, 020021 Bucharest, Romania; 3 Department of Science and Engineering of Oxide Materials and Nanomaterials, Faculty of Chemical Engineering and Biotechnologies, National University of Science and Technology Politehnica Bucharest, 011061 Bucharest, Romania; getav2001@yahoo.co.uk (G.V.); adrian.nicoara@upb.ro (A.-I.N.); 4 Faculty of Material Science and Engineering, National University of Science and Technology Politehnica Bucharest, 011061 Bucharest, Romania; f_miculescu@yahoo.com (F.M.); ciocoiurobert@gmail.com (R.C.)

**Keywords:** dental zirconia, surface treatments, phase transformation, structure changes, compressive strength, biomaterials aging

## Abstract

This in vitro study aimed to compare the effects of various surface treatments and hydrothermal aging on the phase composition, microstructure, and compressive strength of dental zirconia (ZrO_2_). Forty-eight zirconia cubes (8 × 8 × 8 mm) were fabricated using CAD/CAM from two materials: infrastructure zirconia (Group S1) and super-translucent multilayered monolithic zirconia (Group S2). Four samples of each material were analyzed in their pre-sintered state (S1-0, S2-0). The remaining specimens were sintered and assigned to sub-groups based on surface treatment: untreated, sandblasted with 30 µm or 50 µm Al_2_O_3_, polished, or polished and glazed. Characterization was performed using EDX, SEM, XRD with Rietveld refinement, Raman spectroscopy, and compressive testing before and after accelerated hydrothermal aging, according to EN ISO 13356:2015. EDX revealed a higher yttria content in monolithic zirconia (10.57 wt%) than in infrastructure zirconia (6.51 wt%). SEM images showed minimal changes in polished samples but clear surface damage after sandblasting, which was more pronounced with larger abrasive particles. XRD and Raman confirmed that sandblasting promoted the tetragonal (t-ZrO_2_) to monoclinic (m-ZrO_2_) phase transformation (t→m), amplified further by hydrothermal aging. The polished groups showed greater phase stability post-aging. Compressive strength decreased in all treated and aged samples, with monolithic zirconia being more affected. Polished samples displayed the best surface quality and structural resilience across both materials. These findings underline the impact of clinical surface treatments on zirconia’s long-term mechanical and structural behavior.

## 1. Introduction

The evolution of digital systems and automated technologies used in the manufacturing of dental prosthetic restorations has significantly transformed clinical workflows and broadened treatment options for both clinicians and dental technicians, as well as for the patients. In parallel, continuous developments in dental materials have led to ceramic systems with improved biomechanical properties, controlled wear behavior, and superior aesthetics [1]. Zirconia has gained widespread use in dentistry due to its high strength, biocompatibility, and visual resemblance to natural dental structures. It is routinely indicated for a variety of restorations, such as frameworks, veneered or monolithic posterior crowns, anterior and posterior single crowns, anterior three-unit bridges, inlays, onlays, and veneers [2,3,4]. Over the years, multiple zirconia formulations have been introduced, each optimized for different clinical indications and requirements [5].

Despite its advantages, zirconia remains a material whose long-term stability is influenced by a complex interplay of factors. Beyond its chemical composition, which typically includes stabilizing oxides, like yttrium, cerium, magnesium, or calcium, its phase stability also depends heavily on the processing method (subtractive or additive) and on post-processing surface treatments. Increasing the concentration of stabilizing oxides has been shown to enhance translucency, an essential aesthetic requirement, particularly in anterior restorations [5,6,7]. Super-translucent 4Y-, 5Y-, or 6Y-TZP/ tetragonal zirconia polycrystal (containing 4, 5, or 6 mol% Y_2_O_3_) zirconia have high Y_2_O_3_ levels, which increase cubic phase content and reduce the tetragonal phase; this limits t→m transformation, with a reduction in fracture toughness and strength [5]. Following fabrication, zirconia restorations commonly undergo surface modifications to improve adhesion—either to dental hard tissues or to metallic abutments in implant-supported restorations [8,9]. However, these treatments can alter the structural integrity of the material, potentially affecting its long-term behavior and resistance in the oral environment [10]. Moreover, intraoral conditions, particularly exposure to heat and moisture over time, can lead to phase degradation in zirconia, a phenomenon known as aging. This transformation from the tetragonal to the monoclinic phase compromises mechanical properties and can contribute to microcracking and failure over time [11,12]. The selection of prosthetic materials plays a key role in the long-term success of dental restorations supported by natural teeth or dental implants [3,5]. Among zirconia-based materials, two main approaches have emerged: monolithic zirconia restorations, milled entirely from a single block, and multilayer monolithic zirconia blocks, composed of continuous layers with varying translucency, shade, and strength, optimized for CAD/CAM processing. Monolithic zirconia offers high fracture resistance and eliminates the risk of chipping or delamination commonly associated with veneering ceramics; advances in translucency and surface finishing have further enhanced its esthetics and wear behavior [8,10]. Multilayer zirconia blocks provide improved esthetic outcomes due to their gradual transition in optical and mechanical properties, making them particularly suitable for anterior and posterior monolithic restorations [8,10]. Given their different clinical applications, the choice between available zirconia materials should be guided by multiple factors, including biomechanical and esthetic considerations. Investigating the behavior of these zirconia types under various surface treatment protocols, along with the effects of hydrothermal accelerated aging, could offer valuable insights into their long-term stability and performance under simulated oral conditions. Given these concerns, this study aimed to investigate, under in vitro conditions, the effects of common surface treatments used in adhesive cementation protocols on the phase structure and mechanical behavior of two types of CAD/CAM-milled zirconia. The goal was to assess how different treatments, combined with accelerated hydrothermal aging, influence surface morphology, phase stability, and compressive strength [13,14]. We hypothesized that surface treatments involving abrasion—particularly sandblasting with larger alumina particles—would accelerate the tetragonal-to-monoclinic phase transformation and reduce the mechanical integrity of zirconia, more notably in highly translucent materials.

### Purpose of the Study

The purpose of this in vitro study was to evaluate how different surface treatments—commonly used in clinical adhesive cementation protocols—affect the phase composition, surface morphology, and compressive strength of two types of CAD/CAM-milled zirconia: one intended for prosthetic infrastructures and the other for monolithic restorations. Additionally, the study investigated how these treatments interact with hydrothermal aging conditions that simulate long-term oral exposure. By comparing changes in phase transformation, microstructure, and mechanical performance across treated and untreated groups, the research aimed to identify which protocols preserve zirconia’s stability and which may compromise its clinical longevity.

## 2. Materials and Methods

### 2.1. Analyzed Materials

Two types of yttria-stabilized tetragonal zirconia polycrystal (3Y-TZP) materials [5] were used in this study, both supplied by IMES-ICORE GmbH (Eiterfeld, Hessen, Germany).

- CORiTECH Zr is used for dental prosthetic infrastructures (Group S1).
- CORiTECH Zr transpa is a super-translucent multilayered monolithic zirconia that is recommended for monolithic restorations (Group S2).

Detailed specifications, including lot numbers, production and expiration dates, and disc dimensions, are presented in Table 1.

According to the manufacturer’s recommendations, zirconia called CORiTECH Zr (IMES-ICORE GmbH, Eiterfeld, Hessen, Germany) is recommended for dental prosthetic restoration infrastructures, including up to 16 units, having very good mechanical properties, biocompatibility, good mechanical strength, and resistance to aging [15,16].

The material called CORiTECH Zr transpa (IMES-ICORE GmbH, Eiterfeld, Hessen, Germany) is, according to the manufacturer’s specifications, represented by pre-colored and translucent zirconium dioxide (zirconia/ZrO_2_) [15,16,17]. The material is recommended for monolithic prosthetic restorations, including up to 16 units. It is characterized as biocompatible, resistant to aging, having outstanding mechanical qualities, and having very good adaptation [15,16,17]. 3Y-TZP represents an earlier generation of zirconia ceramics, and more recent developments have led to the availability of materials with higher yttria content, such as 4Y-PSZ and 5–6Y-PSZ, which offer improved translucency but reduced mechanical strength. The decision to employ 3Y-TZP in our study was based on its well-documented mechanical reliability and its continued clinical relevance in high-stress applications, particularly in posterior restorations.

### 2.2. Sample Design and CAD/CAM Manufacturing

The zirconia samples were subtractively processed with the help of CAD/CAM technology (“Computer-Aided Design/Computer-Aided Manufacturing”). The CAD/CAM technology used in dentistry includes, in summary, data acquisition (e.g., intra- and/or extraoral scanning), processing of acquired data (with specific software e.g. Exocad, Blender for dental), and actual manufacture of the finished product (by subtractive or additive processes) [18]. The samples tested in this study were made by the subtractive method (computerized milling), which was applied properly, respecting the specifications and recommendations of the manufacturer of these materials.

The virtual project of the zirconia samples was made considering the final dimensions of the samples and the degree of shrinkage of the material in the sintering stage. The software generates an .stl file, which has been sent to the milling machine. The automatic positioning of the samples at the level of the zirconia disc was followed by the verification of the correctness of their position and the support pins. The zirconia disc was fixed in the milling machine, and then the computerized milling process could be started.

In this scientific research, zirconia discs with a diameter of 98 × 14 mm (IMES-ICORE GmbH, Eiterfeld, Hessen, Germany) and, respectively, Zr transpa with the same diameter (IMES-ICORE GmbH, Eiterfeld, Hessen, Germany), presented above, in Table 1, were used. A total of 48 pre-sintered zirconia samples (24 of each material) were designed and milled using CAD/CAM technology. Each sample was initially designed as a 10 mm cube, considering the material’s sintering shrinkage factor. Of the 48 samples, 40 were subsequently subjected to sintering (20 samples for each material). After sintering, the tests were dimensionally similar, with a cube side of 8 mm.

The .stl files were generated and milled using a five-axis Planmeca PlanMill 60 S milling machine (Planmeca, Helsinki, Finland). Subsequently, after the completion of the computerized milling, the zirconia samples were removed from the level of the zirconia disc by cutting/sectioning the support pins. The samples were then minimally finished by reducing the excess areas remaining from the support pins and by smoothing the minimum finishing of these areas. These interventions on the zirconia samples were performed according to the manufacturer’s recommendations, the processing being carried out with abrasive tools, such as red ring diamond stones, by the same qualified, experienced dental technician.

### 2.3. Sintering Process

Sintering was performed using the Programat S2 furnace (Ivoclar Vivadent GmbH, Schaan, Liechtenstein), following the manufacturer’s instructions [15]. The process included homogeneous heating up to 1600 °C, resulting in final cubic samples of 8 mm per side. The sintering step induced an estimated 20% volume reduction.

Sintering furnace Scheduled S2 (Ivoclar Vivadent GmbH, Schaan, Liechtenstein) is modern, ergonomic, and compact, with excellent performance due to optimized software. The furnace’s sintering programs are coordinated with the range of zirconium dioxide materials and allow the choice of a series of programming options, thus achieving an appropriate final strength of the material and a very good accuracy of the final parts [19].

### 2.4. Surface Treatments

The two initial zirconia groups—S1 (infrastructure zirconia/(CORiTECH Zr) and S2 (monolithic zirconia/CORiTECH Zr transpa)—were divided into specific sub-groups, depending on the interventions performed on their surface, and coded accordingly.

All the interventions on the zirconia surfaces were performed by the same qualified, experienced dental technician, according to the manufacturer’s instructions. The details regarding the interventions carried out on the surfaces of the zirconia samples are presented in summary below.

The 1st sub-group, represented by pre-sintered zirconia, contained 4 samples for each material (coded S1-0 and S2-0, respectively) and had no intervention on the surface.

The 2nd sub-group, represented by sintered zirconia, contained 4 samples for each material (coded S1-1 and S2-1, respectively). There was no intervention on the surface of these samples.

The 3rd sub-group, represented by sintered and sandblasted zirconia, contained 8 samples for each material and 16 samples in total. For each material, this sub-group was divided into two other mini-groups; the first mini-group (coded S1-2a and S2-2a, respectively), containing 4 samples each, being sandblasted with aluminum oxide with a particle size of 30 μm (CoJetTM Sand, Blast Coating Agent 30 μm, 3M ESPE, St. Paul, MN, USA), and the second group (coded S1-2b, and S2-2b, respectively), containing 4 samples each), being sandblasted with aluminum oxide with a particle size of 50 μm (aluminum oxide 50 μm for intraoral sandblaster, OrthoFocus). For the rest, the sandblasting of the zirconia surfaces was carried out in compliance with the details of the following protocol: sandblasting distance—10 mm; pressure used for sandblasting—2 bar (29 psi); sandblasting angle—between 75 and 900; and sandblasting maintenance time—between 5 and maximum 10 s.

The 4th sub-group, represented by sintered and hand-polished zirconia, contained 4 samples for each material and 8 samples in total (coded S1-3 and S2-3, respectively). The sintered zirconia samples were polished according to the manufacturer’s instructions using the system/set/kit: OptraGloss Ceramic Kit, Ivoclar Vivadent GmbH, Schaan, Liechtenstein. The system is composed of two types of tools: diamond tools for pre-polishing (finishing), called PP, dark blue in color, and diamond tools for high-gloss polishing, called HP, in light blue color. The manufacturer recommends pre-polishing with dark blue gums and then polishing for high gloss with light blue gums [5]. The OptraGloss Ceramic Kit polishing system (Ivoclar Vivadent GmbH, Schaan, Liechtenstein) is recommended by the manufacturer for lithium disilicate, zirconia, leucitic ceramics, and composite resins [5].

The 5th sub-group, represented by sintered, hand-polished, and glazed zirconia, contained 4 samples for each material and 8 samples in total (coded S1-4 and S2-4, respectively). Sintered zirconia samples have been polished using the same polishing set mentioned above (OptraGloss Ceramic Kit, Ivoclar Vivadent GmbH, Schaan, Liechtenstein) and following the same steps recommended by the manufacturer: pre-polishing and polishing for intense gloss. Subsequently, the samples were cleaned in an ultrasonic bath, in alcohol, after which the glazing stage was carried out in the Scheduled P510 (Ivoclar Vivadent GmbH, Schaan, Liechtenstein) following the manufacturer’s recommendations.

All sub-groups of the zirconia samples mentioned above are presented in Table 2.

Table 3 also summarizes the peculiarities of the sintered zirconia samples included in this study and the surface treatments performed.

### 2.5. Accelerated Aging Protocol

Half of the sintered and superficially prepared samples were subjected to the accelerated hydrothermal aging test according to the EN ISO 13356-2015 standard [6] for specific ceramic materials based on yttria stabilized tetragonal zirconia. The hydrothermal treatment was carried out by immersing the samples in water using sealed containers (autoclaves), with small volumes, on 3 cycles (C): C1-5 h, C2-10 h, and C3-25 h at a temperature of 134 °C and a pressure of 2–3 bar. According to the standard (EN ISO 13356-2015 [6]), each cycle can be equated to about 4–5 years of “in vivo” use. Each sample was analyzed beforehand and after the hydrothermal treatment of each cycle, compositionally and morphologically by electron microscopy (EDAX-SEM) and microstructurally by X-ray diffraction and RAMAN spectroscopy.

### 2.6. Analytical Methods

The analyses and experimental tests in this study were carried out at the Faculty of Chemical Engineering and Biotechnologies, Department of Science and Engineering of Oxide Materials and Nanomaterials, and the Faculty of Materials Science and Engineering, Electron Microscopy and Microanalysis Laboratory, Polytechnic University of Bucharest.

All samples were analyzed and tested with the following:

1. X-ray spectroscopy (EDX);
2. Scanning electron microscopy (SEM);
3. X-ray diffraction and Rietveld processing of diffractograms;
4. RAMAN spectroscopy;
5. Compression test.

X-ray diffraction analysis (XRD) and scanning electron microscopy (SEM) are procedures used to investigate the chemical composition, morphology, and size of zirconia crystals. X-ray diffraction provides information about the crystal structure of zirconia (type of crystal lattice, interatomic distances, orientation of crystals), and the Rietveld refinement assesses the mineralogical composition. For these, a PANalytical Empyrean (Almelo, The Netherlands) diffractometer provided with a characteristic Cu X-ray tube (λ CuKα1 = 1.541874 Å) was used, and the samples were scanned in the 2θ angle range of 20–80° with a scan increment of 0.02° and a time of 100 s/step. SEM analyses were performed with an Inspect F-50 high-resolution scanning electron microscope (SEM) coupled with an energy-dispersive X-ray spectroscopy (EDX) detector (Thermo Fisher—former FEI, Eindhoven, The Netherlands); they allow visualization on a microscopic scale of the morphology and size of zirconia crystals. SEM uses a beam of electrons to obtain three-dimensional images of the surface of materials.

Both pre-sintered and sintered samples were compositionally characterized by X-ray diffraction and RAMAN spectroscopy. Raman spectroscopy was performed with a Horiba Confocal LabRAM HR Evolution spectrophotometer (Horiba, Kyoto, Japan) using a 633 nm laser, a 100% ND filter, 50 × objective, and 600 g/mm grating; the wavenumber ranged between 50 and 800 cm^−1^ and there were 10 scans per sample and 10 s per scan.

After these analyses, the samples were subjected to pure compression testing (vertical compression) with the help of a Walter + Bai LFV 300 universal testing machine with a capacity of 300 kN and controllable test parameters. In this case, the test speed was 1 mm/minute.

## 3. Results

### 3.1. Chemical Composition (EDX Analysis)

**Figure 1:**
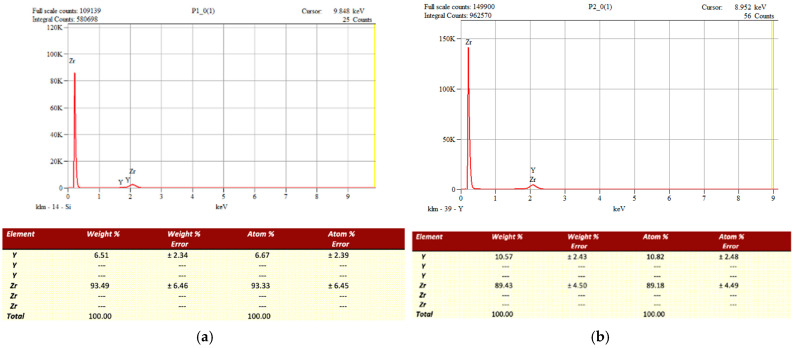
EDX analysis of the two materials taken in the study. (**a**) EDAX analysis of pre-sintered samples from CORiTECH Zr (IMES-ICORE GmbH, Eiterfeld, Hessen, Germany); (**b**) EDAX analysis of CORiTECH Zr transpa pre-sintered samples (IMES-ICORE GmbH, Eiterfeld, Hessen, Germany).

The chemical composition of the two pre-sintered zirconia materials was evaluated using Energy Dispersive X-ray Spectroscopy (EDX). The analysis revealed similar elemental profiles, with zirconium and oxygen being predominant. However, a notable difference in yttria (Y_2_O_3_) content was observed between the two materials (Figure 1).

CORiTECH Zr transpa, intended for monolithic restorations, showed a higher yttria content (10.57 wt%) compared to CORiTECH Zr, which is designed for prosthetic infrastructures (6.51 wt%). This increase in yttria concentration in the monolithic zirconia is associated with improved translucency, enhancing its aesthetic properties [5,7].

### 3.2. Surface Morphology (SEM Analysis)

**Figure 2:**
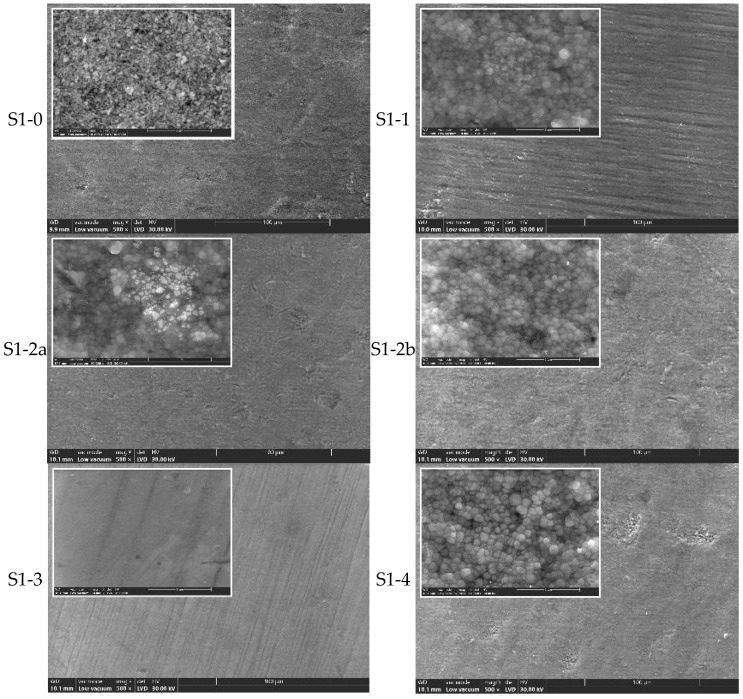
SEM images for zirconia S1-0, S1-1, S1-2a, S1-2b, S1-3, and S1-4 (×500, ×10k).

**Figure 3:**
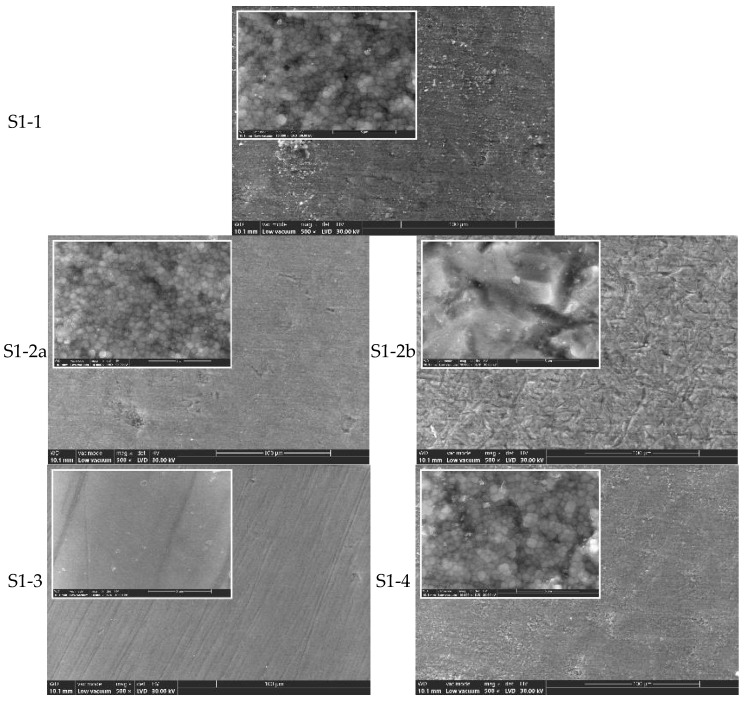
SEM images for zirconia S1-1, S1-2a, S1-2b, S1-3, and S1-4, processed by different methods and hydrothermally treated at 134 °C/2–3 bar, 25 h (×500, ×10k).

**Figure 4:**
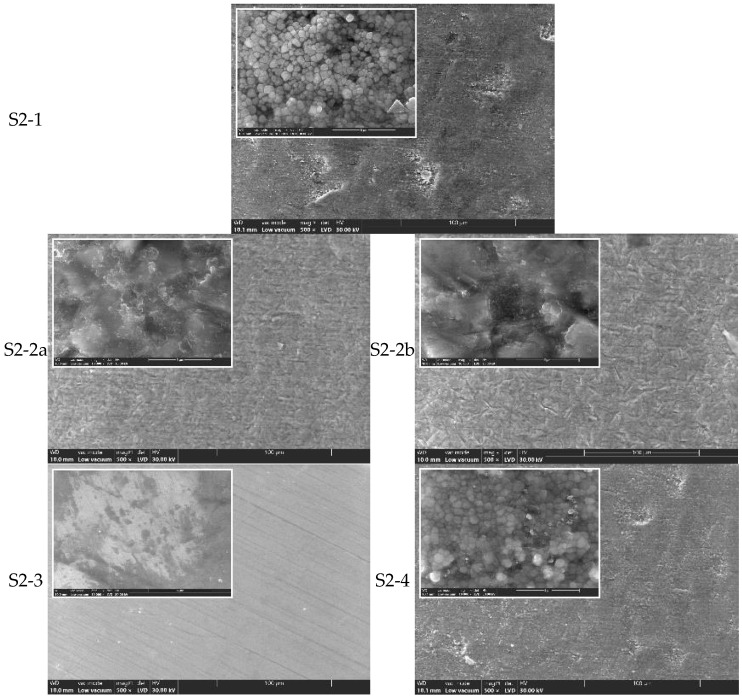
SEM images for zirconia S2-1, S2-2a, S2-2b, S2-3, and S2-4 (×500, ×10k).

**Figure 5:**
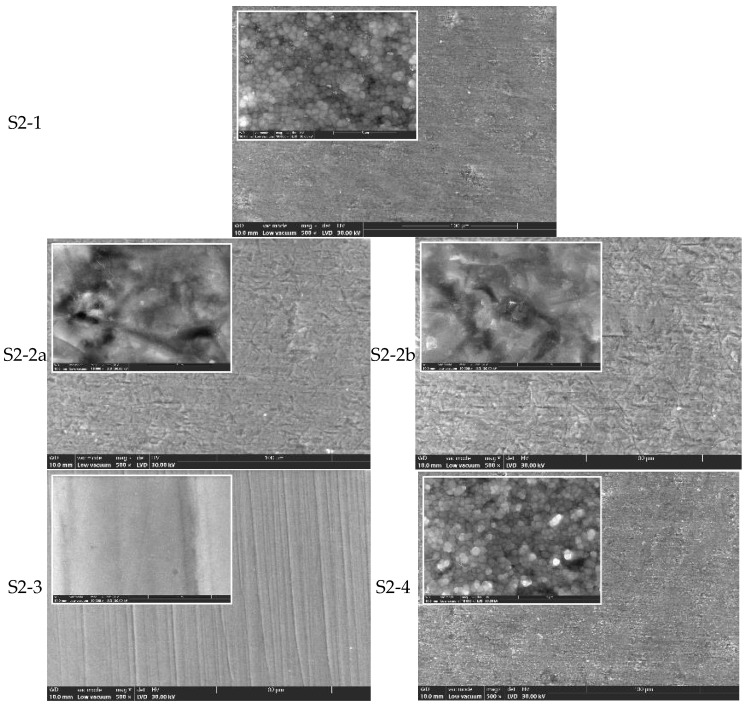
SEM images for zirconia S2-1, S2-2a, S2-2b, S2-3, and S2-4 processed by different methods and hydrothermally treated at 134 °C/2–3 bar, 25 h (×500, ×10k).

Scanning electron microscopy (SEM) was used to examine the surface morphology of all zirconia samples (S1 and S2) processed by different methods, before and after hydrothermal treatment (134 °C/2–3 bar, 25 h) (Figure 2, Figure 3, Figure 4 and Figure 5).

SEM observations of the S1 series (CORiTECH Zr) (Figure 2):

S1-0 (pre-sintered) exhibited a porous microstructure composed of small zirconia grains (d ≤ 0.25 µm).

S1-1 (sintered, untreated) showed a denser structure with grain junctions indicating complete sintering.

S1-2a and S1-2b (sandblasted) presented surface damage, such as microcracks and craters, which were more pronounced with 50 µm alumina particles (S1-2b).

S1-3 (polished) displayed the smoothest surface, with no significant defects.

S1-4 (polished and glazed) is a smooth surface with isolated rough patches, likely induced by thermal glazing effects.

SEM observations of the S2 series (CORiTECH Zr transpa) (Figure 4):

For S2-1 to S2-4, similar trends were observed, though S2 groups generally showed more extensive surface damage post-sandblasting compared to S1. SEM images indicated that monolithic zirconia is more sensitive to abrasive processing. The polished monolithic samples (S2-3) maintained the most uniform and defect-free surfaces.

In Figure 2, commercial pre-sintered ceramics (S1-0) are characterized by a porous microstructure, made up of small zirconia particles (d ≤ 0.25 μm). Through sintering heat treatment, zirconia densifies, the particles increasing in size, reaching triple junction points (maximum densification).

Moreover, from the analysis of the SEM images, it results that by sandblasting surface defects, cracks and craters are induced (Figure 2 and Figure 4), which most likely promote the transformation of the tetragonal (t-ZrO_2_) to monoclinic (m-ZrO_2_) phase due to the energy involved. This phenomenon appears to be more pronounced for S2 zirconia. It is found that this phenomenon is also accentuated by the increase in the size of the alumina particles with which the sandblasting is performed.

The samples processed by manual polishing have the finest quality surface, as shown inFigure 2 (S1-3) and Figure 4 (S2-3), followed by the only sintered and unprocessed samples, respectively, and the glazed ones, which are the white arrows in Figure 2 (S1-1 and S1-4) and Figure 4 (S2-1 and S2-4). On the surface of the latter, there are areas where the t→m transformation (white arrows) has been favored, and this is most likely an effect of the cooling treatment.

Post-Aging SEM Observations (Figure 3 and Figure 5):

Hydrothermal aging at 134 °C/2–3 bar for 25 h induced further surface degradation in sandblasted samples (S1-2a, S1-2b, S2-2a, S2-2b), with increased microcrack formation and monoclinic phase grains visible at high magnification. In contrast, polished samples (S1-3 and S2-3) showed minimal structural changes post-aging, indicating higher resistance to low-temperature degradation.

The hydrothermal treatment at 134 °C/2–3 bar for 25 h favors the t-m transformation, the most affected being samples S1-2a and S1-2b (Figure 3) and samples S2-2a and S2-2b (Figure 5) (at high magnification, the presence of large m-grains and microcracks is observed). The best behavior in terms of degradation at low temperature is samples S1-3 (Figure 3) and S2-3 (Figure 5), for which there are no important microstructural changes when compared to S1-3 (Figure 2) and P2-3 (Figure 4).

### 3.3. Phase Composition (XRD and Rietveld Analysis)

**Figure 6:**
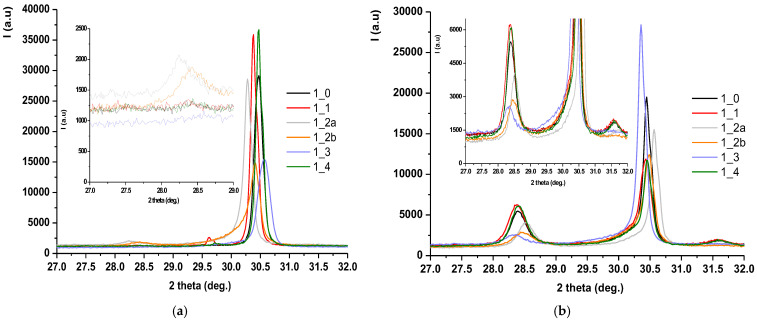
X-ray diffraction for S1-coded zirconia samples processed by different methods (**a**) and hydrothermally treated at 134 °C/2–3 bar, 25 h (**b**).

X-ray diffraction (XRD) patterns, followed by Rietveld refinement, were used to quantify the relative amounts of tetragonal (t-ZrO_2_) and monoclinic (m-ZrO_2_) phases before and after hydrothermal treatment. Figure 6 shows the diffractograms for zirconia samples coded S1 processed by different methods: (a) untreated hydrothermally and (b) treated hydrothermally at 134 °C/2–3 bar for 25 h.

Pre-Aging Observations:

Untreated sintered samples (S1-1, S2-1) were predominantly in the tetragonal phase (>98%).

Sandblasted samples showed a significant increase in monoclinic phase, more so with 50 µm particles. For instance, S2-2a, 68.5% t-ZrO_2_/31.5% m-ZrO_2_, and S2-2b, 56.2% t-ZrO_2_/43.8% m-ZrO_2_.

Post-Aging Results:

Hydrothermal aging accelerated the t→m transformation across all treated samples.

The most affected groups were S2-2a and S2-2b, where degradation led to additional monoclinic phase formation.

In contrast, polished groups (S1-3 and S2-3) maintained higher tetragonal content after aging; for example, S2-3 after aging was 84.1% t-ZrO_2_/15.9% m-ZrO_2_.

Rietveld analysis also showed an erratic monoclinic content variation in S2 sandblasted groups, possibly due to surface detachment of m-ZrO_2_ grains after the first aging cycle.

**Figure 7:**
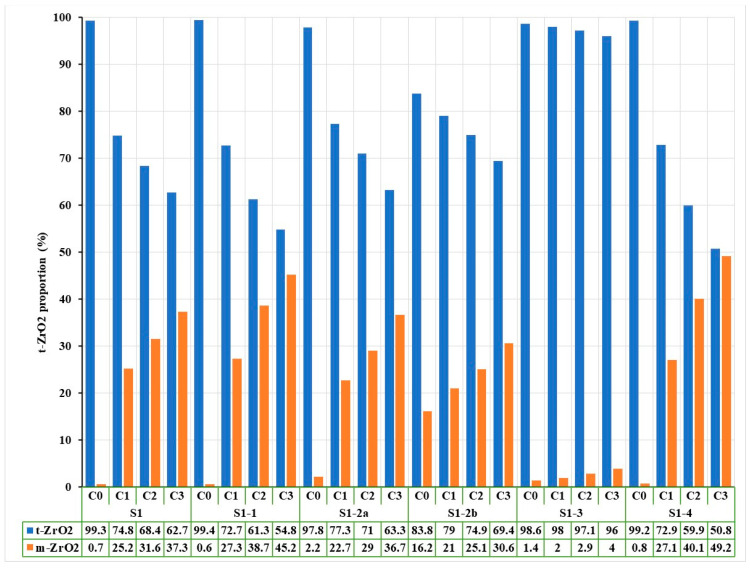
Variation of phase composition as a function of hydrothermal treatment duration by Rietveld processing of X-ray diffraction results for zirconia samples encoded S1 processed by different methods and hydrothermally treated at 134 °C/2–3 bar for three cycles (C1-5 h, C2-10 h, C3-25 h).

**Figure 8:**
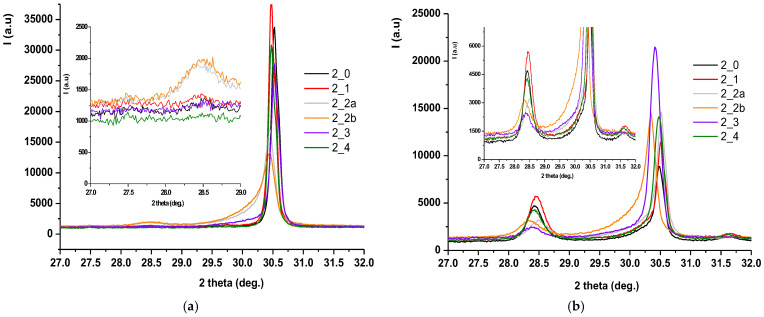
X-ray diffraction for S2-encoded zirconia samples processed by different methods (**a**) and untreated and hydrothermally treated at 134 °C/2–3 bar, 25 h (**b**).

**Figure 9:**
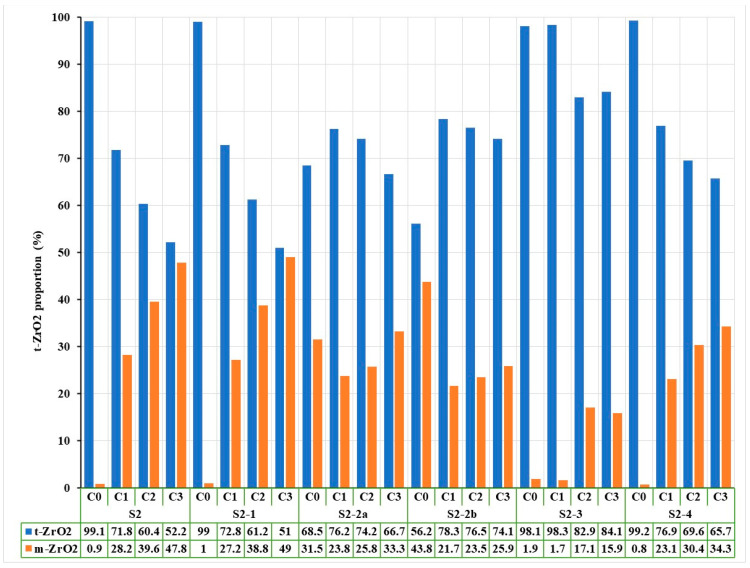
Variation of phase composition as a function of hydrothermal treatment duration by Rietveld processing of X-ray diffraction results for S2-encoded zirconia samples processed by different methods and hydrothermally treated at 134 °C/2–3 bar for three cycles (C1-5 h, C2-10 h, C3-25 h).

Also, Figure 7 shows the Rietveld processing of diffractograms performed on untreated and hydrothermally treated zirconia samples at 134 °C/2–3 bar for three cycles (H1-5 h, H2-10 h, H3-25 h), according to EN ISO 13356-2015. Similarly, for the zirconia samples encoded in S2, the X-ray diffraction results were presented, processed, and then rendered in Figure 8 and Figure 9.

For both types of sintered zirconia, the subsequent sandblasting processing procedure induced the formation of monoclinic zirconia (PDF 00-065-0687) (Figure 6a and Figure 8a, 2 theta in the range of 28–29 deg.), and the larger the size of the alumina particles is important (Figure 7, comparison between S1-2a, C0 and S1-2b, C0, respectively, and Figure 9, comparison between S2-2a, C0 and S2-2b, C0). Thus, it can be considered that the abrasion/impact energy is high enough for the tetragonal granules to take it up and pass into the monoclinic form.

It can also be seen that for all materials, the application of hydrothermal treatment at 134 °C/2–3 bar favors the transformation of the tetragonal form (PDF 01-080-2187) into the monoclinic form (PDF 00-065-0687), as show in Figure 6b and Figure 8b, a process that is intensified by the increase in the hydrothermal heat treatment period, as shown in Figure 7 and Figure 9.

A special situation is recorded for samples S2-2a and S2-2b, for which it is observed that there is a random variation of the amount of monoclinic phases with the number of hydrothermal cycles applied. This can be explained by the fact that these samples, even after the first cycle of hydrothermal treatment, undergo an advanced degradation process, which favors the detachment of monoclinic particles from the surface of the material.

Figure 7 and Figure 9 show that the best structural stability is presented by S1-3 and S2-3.

From the point of view of stability/durability for the two types of materials tested (zirconia S1 and zirconia S2), it results that zirconia S1 is much more stable than zirconia S2, with the amount of monoclinic phases formed in zirconia S2 being much higher than in zirconia S1, as shown by the comparison between Figure 7 and Figure 9.

### 3.4. Raman Spectroscopy

**Figure 10:**
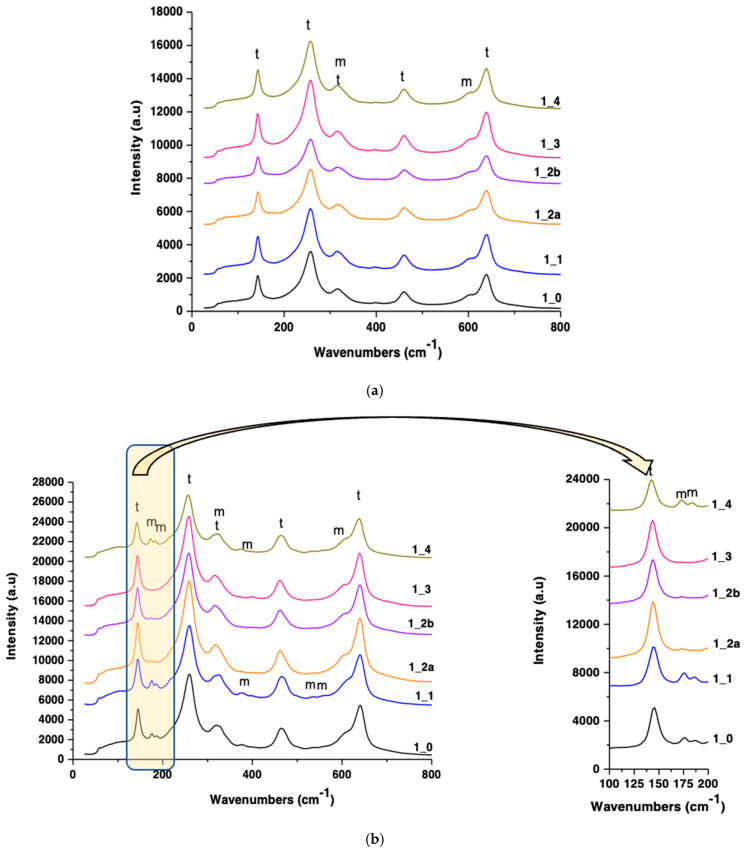
RAMAN spectra for S1-coded zirconia samples processed by different methods (**a**) and hydrothermally treated at 134 °C/2–3 bar, 25 h (**b**) (tetragonal zirconia—t, monoclinic zirconia—m).

**Figure 11:**
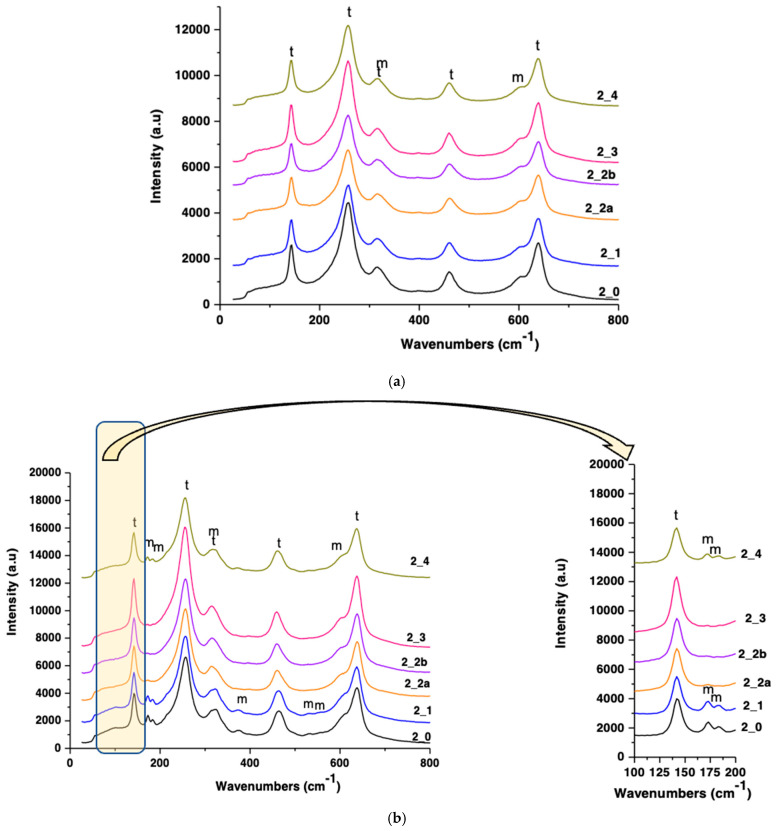
RAMAN spectra for S2 zirconia samples processed by different methods (**a**) and hydrothermally treated at 134 °C/2–3 bar, 25 h (**b**) (tetragonal zirconia—t, monoclinic zirconia—m).

Raman analysis confirmed the XRD findings (presented in Figure 6, Figure 7, Figure 8 and Figure 9). Tetragonal zirconia displayed characteristic vibrational bands at 142, 257, 324, 461, and 639 cm^−1^, while monoclinic zirconia bands appeared at 172, 184, 376, 529, and 603 cm^−1^ (Figure 10 and Figure 11).

S1 and S2 sandblasted samples showed strong monoclinic signals, particularly post-aging.

Polished samples retained dominant tetragonal peaks with minimal monoclinic presence.

### 3.5. Mechanical Properties (Compression Testing)

**Figure 12:**
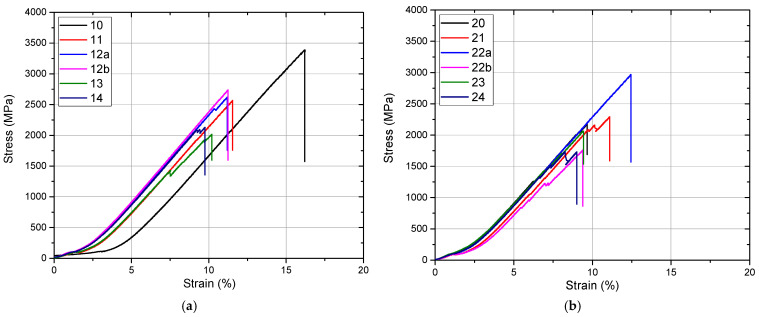
Tension–strain curves of the two types of zirconia materials: S1 (**a**) and S2 (**b**).

After specific surface preparations, half of the specimens were subjected to compression. The results of the compression analysis of the two categories of samples (S1 and S2) analyzed before being subjected to the accelerated aging process are presented in the graphs in Figure 12.

Mechanical compression testing showed that both materials have a brittle behavior with no plastic deformation, which fails immediately after leaving the linear elastic region. Also, for both materials, it has been observed that cracks are primed before breaking.

Although both materials meet clinical use parameters, there are significant differences in compressive strength, with the more transparent zirconia material with a higher yttrium oxide load being less resistant according to our mechanical evaluations. For both types of zirconia ceramics, the surface treatments performed significantly negatively and influenced the mechanical strength of the tested samples.

**Figure 13:**
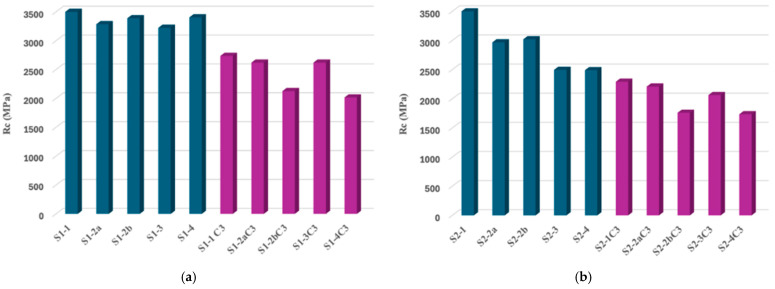
Compressive strength values of the two types of zirconia materials, S1 (**a**) and S2 (**b**), subjected to different surface treatments without and with accelerated aging hydrothermal treatment.

The second half of the prepared samples were first subjected to the three hydrothermal cycles of accelerated aging and then were subjected to the compression test. The compressive strength values of all samples can be seen in Figure 13.

The results of the mechanical compressive strength test confirmed the imaging observations obtained by SEM microscopy as well as the diffractometric results (Figure 13). In both cases of zirconia for infrastructure and zirconia for monolithic restorations, the mechanical strength of the samples without surface treatment was the maximum. Although the values of the mechanical strength between the two samples of sintered and unprepared zirconia materials (S1-0 and S2-0, Figure 13a,b) are similar, the surface treatment of ceramics for monolithic restorations determined a significant reduction in their compressive strength. After hydrothermal treatment of accentuated aging, the compressive strength values decreased for both types of zirconia materials, with a dramatic decrease observed in all the samples obtained from zirconia for monolithic restorations.

#### Compression Testing Before and After Aging

Pre-Aging Compression Test

The compression stress–strain behaviour of all zirconia samples indicated a characteristic brittle fracture with the absence of any plastic deformation. Crack initiation occurred prior to the final failure, which is consistent with the known mechanical response of ceramic materials. Among the tested groups, the highest compressive strength was observed in the sintered, untreated specimens (S1-1 and S2-1), suggesting that surface integrity plays a critical role in maintaining mechanical performance. In contrast, the lowest compressive strength was recorded for the sandblasted monolithic zirconia sample (S2-2b), highlighting the detrimental effect of surface treatments that introduce flaws. Polished samples retained relatively higher strength values compared to other treated groups, indicating that surface smoothness contributes positively to the mechanical integrity of zirconia prior to aging.

Post-Aging Compression Test

Following hydrothermal aging, all groups exhibited a reduction in compressive strength. This degradation was more pronounced in the monolithic zirconia samples belonging to the S2 series compared to the infrastructure zirconia (S1 series), reflecting the greater susceptibility of monolithic zirconia to low-temperature degradation. Among all tested conditions, the S2-2b sample (sandblasted with 50 µm alumina particles) showed the most significant mechanical deterioration post-aging. Conversely, the polished samples (S1-3 and S2-3) demonstrated the highest compressive strength after aging, underscoring the protective effect of surface polishing in mitigating aging-induced degradation.

Image Analysis and relation with Mechanical Properties

The average grain size determined using the Heyn method is 0.41 ± 0.02 µm. It does not vary significantly between sets or states, and cycling does not influence the grain size. This would be an additional observation for the SEM analysis.

A roughness analysis performed based on grayscale levels in SEM images at 1000× magnification indicates that the surface is significantly affected by the applied treatment.

In set S1, there is a tendency for roughness to increase after cycling (apart from sample 13), while in set S2, roughness tends to decrease (except for samples 21 and 23).

**Figure 14:**
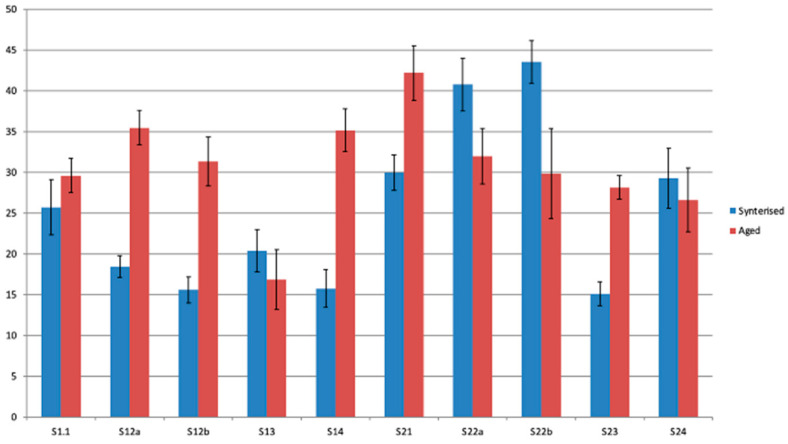
The average grain size measurement (Heyn method analysis).

Another relation appears between the percentage of the monoclinic phase and compressive strength, with a correlation coefficient of 0.66 for S1 and 0.56 for S2. There is also a strong correlation between roughness and compressive strength, with a correlation coefficient of 0.74 for S1 and 0.75 for S2. If we consider the percentage of monoclinic phase and roughness as independent factors, compressive strength would be largely dictated by surface defects, acting as initiation points for fracture (Figure 14).

## 4. Discussion

The scientific research carried out within this study focused on the analysis of the influence that certain surface treatments of zirconia have on the phase composition and microstructure and, implicitly, on the compressive strength of this dental biomaterial. In order to quantify the impact of these surface treatments on the long-term clinical success of restorations obtained from these materials, the hydrothermal method of accelerated aging of dental zirconia was used according to EN ISO 13356-2015 [6]. The results of our study could provide a better understanding of the durability and performance of these biomaterials in simulated oral environments. Such data can support clinicians in making informed decisions regarding the selection of prosthetic materials, ultimately guiding restorative protocols and improving patient outcomes.

The materials included in the study were zirconia used as infrastructure for prosthetic restorations (CORiTECH Zr, IMES-ICORE GmbH, Eiterfeld, Hessen, Germany) as well as monolithic zirconia (CORiTECH Zr transpa, IMES-ICORE GmbH, Eiterfeld, Hessen, Germany). Zirconia ceramics stabilized with different concentrations of yttria (Y_2_O_3_) exhibit distinct property profiles. 3Y-TZP (3 mol% yttria-stabilized tetragonal zirconia polycrystal) is characterized by high flexural strength and fracture toughness, owing to the transformation toughening mechanism of the tetragonal phase. In contrast, materials with higher yttria content, such as 4Y-PSZ and 5–6Y-PSZ, contain increasing amounts of the cubic phase, which enhances translucency but compromises mechanical strength and resistance to crack propagation. Given the aims of our study, which focus on the influence of specific surface treatments on the phase composition, microstructure, and compressive strength of zirconia, 3Y-TZP was selected as the most appropriate material. Its inclusion ensures relevance to clinical scenarios requiring high strength, particularly in load-bearing regions, and could support the generation of robust experimental data. From both types of materials, samples were obtained by CAD/CAM technology that were treated differently on the surface and grouped accordingly, as follows: pre-sintered zirconia samples (without additional interventions); samples of sintered zirconia (without surface interventions); samples of zirconia sintered and sandblasted with aluminum oxide particles with particle sizes of 30 μm and 50 μm; samples of sintered and hand-polished zirconia; and samples of sintered, hand-polished, and glazed zirconia.

The experimental tests performed in this study were as follows: EDAX analysis, X-ray diffraction and Rietveld processing of diffractograms, RAMAN spectroscopy, and scanning electron microscopy (SEM). X-ray diffraction provided information about the crystal structure of the zirconia analyzed. Materials included in the study have been characterized compositionally (phasic) by X-ray diffraction and RAMAN spectroscopy. On the other hand, scanning electron microscopy (SEM) allowed the micrometer-scale visualization of the size and morphology of zirconia crystals. Also, the materials included in the study were tested for stability in the environment of use by the hydrothermal method, according to the EN ISO 13356-2015 standard [6]. After performing the hydrothermal treatment, the zirconia samples were characterized again, compositionally, using X-ray diffraction analysis and RAMAN spectroscopy, as well as microstructurally (by scanning electron microscopy).

It was observed that, in both types of sintered zirconia, the sandblasting procedure induced the formation of monoclinic zirconia, a phenomenon manifested all the more intensely the larger the size of the alumina particles used in sandblasting. It is possible that the impact energy was large enough for the tetragonal granules to take it up and pass into the monoclinic form.

Also, the hydrothermal treatment at 134 °C/2–3 bar favored the transformation of the tetragonal form into the monoclinic form for all the materials included in the study. In particular, samples of sandblasted translucent monolithic zirconia undergo, even after the first cycle of hydrothermal treatment, an advanced degradation process, which allows the detachment of monoclinic particles from the surface of the material. The best structural stability was shown by the zirconia samples dedicated to the prosthetic restoration infrastructures; the amount of monoclinic phase formed in this material was much lower than that formed in super-translucent multilayered monolithic zirconia. Also, among the types of samples, polished ones, belonging to both materials, had the best structural stability.

The results of scanning electron microscopy were correlated with the results of X-ray diffraction and RAMAN spectroscopy; thus, with SEM electron microscopy, it was found that surface defects, cracks, and craters are generated by sandblasting, which probably induce the transformation of t-ZrO_2_ to m-ZrO_2_, a phenomenon directly proportional to the size of the blasting particles (those sandblasted with 50 μm alumina particles showed more surface defects compared to the those sandblasted with 30 μm alumina particles). Although, with electron microscopy, it was observed that the zirconia samples processed by manual polishing have the smoothest surface qualitatively after sandblasting, regardless of the size of the sandblasting particles, and zirconia intended for monolithic restorations showed more surface defects compared to infrastructural zirconia.

Mechanical compressive strength tests confirmed SEM imaging and diffractometric analytical observations. The graphs of the results of the compression tests recorded were homogeneous, indicating a lower value of the mechanical strength of zirconia for monolithic restorations compared to zirconia for infrastructure, a decrease that is dependent on the type of surface preparation. After hydrothermal treatment of accelerated aging, the lowest mechanical resistance was recorded by the samples obtained from zirconia for monolithic restorations, regardless of their surface treatment.

Mechanical surface treatments.

Numerous studies have explored mechanical methods of zirconia surface modification, including sandblasting and polishing [20,21,22,23]. These techniques are commonly used to improve surface roughness and enhance bonding strength. While sandblasting can increase micromechanical retention, it also promotes surface defects and phase transformation. In contrast, manual polishing has been shown to create smoother surfaces with favorable biological responses.

Photofunctionalization and plasma treatment.

Non-mechanical approaches, such as ultraviolet (UV) photofunctionalization [24,25,26] and cold atmospheric plasma treatment [27,28,29,30], have gained attention for their ability to improve cell adhesion and reduce bacterial colonization on zirconia surfaces. UV treatment can enhance protein adsorption and fibroblast attachment, while plasma applications have demonstrated antibacterial efficacy and improved epithelial sealing.

Surface coatings.

Advanced coating strategies have also been proposed to enhance the biological and mechanical behavior of zirconia. These include titanium dioxide (TiO_2_) [31,32,33], germanium [34,35], and zinc oxide (ZnO) nanocrystal coatings [36]. Such modifications aim to improve soft tissue integration and reduce biofilm formation on zirconia-based restorations.

Effect of glazing versus polishing.

Several studies have compared the mechanical and optical outcomes of glazing versus polishing on monolithic zirconia. The findings suggest that glazing tends to reduce flexural strength, while manual polishing preserves or even improves surface quality and strength [37,38,39,40]. As a result, polishing is often recommended over glazing when mechanical integrity is a priority.

A comparison of our results with those reported in other studies reveals a consistent alignment of the findings. Thus, several scientific studies on mechanical treatments applied to zirconia surfaces [20,21,22,23,30] indicate that in the context in which zirconia is polished by hand, peri-implant tissues have a favorable response, with low bacterial adhesion and reduced epithelial tissue inflammation. Moreover, regarding mechanical surface treatments of zirconia, Maruo et al., 2020 [36] state that the sandblasting of highly translucent multilayered monolithic zirconia (KATANA UTML, Kuraray Noritake Dental, Tokyo, Japan) produces microcracks on the surface, compromising the mechanical behavior of the material. Hammoudeh et al., 2024 [37] confirm that the various surface treatments applied to high translucency zirconia, namely, milling with diamond stones with different grains and sandblasting with 50 μm alumina combined with hydrothermal treatment, favor the transition from the tetragonal phase to the monoclinic phase of zirconia. It is worth noting that sandblasting contributes to a stronger bond between zirconia and the adhesive fixing cement, which may compensate for the deterioration of zirconia’s mechanical properties. On the other hand, Jamali et al., 2024 [38] demonstrated that the polishing of zirconia surfaces (monolithic of various translucencies) improves its surface properties and causes the smallest changes in the mechanical properties of the material compared to other surface treatments (sintering and glazing; sintering and polishing; sintering, glazing and milling; sintering, glazing, milling, and polishing; sintering, glazing, milling, and polishing; sintering, glazing, milling, and polishing). In the same line, Ozturk et al., 2022 [39] conclude that polishing zirconia surfaces can improve the physical properties of the material without affecting its mechanical properties. In this regard, the authors recommend manual polishing of zirconia as a more favorable alternative to glazing, as glazing reduces the bending strength of zirconia. Sarabi et al., 2024 [40] claim that milling zirconium and polishing it increases bending strength, whereas glazing did not produce a significant impairment of this parameter. The study results by Hatanaka et al., 2020 [41] confirm the above, indicating that the glazing of zirconia tends to decrease its flexural strength. Another relevant study on the surface treatment of translucent and super-translucent monolithic zirconia and hydrothermal treatment is that of Vila-Nova et al., 2020 [42], specifying that the monoclinic phase was observed predominantly in hydrothermally treated zirconia samples (127 °C, 1.7 bar/24 h); the authors also recommend manual polishing with silicone gums, which produces the finest of the tested surfaces (polishing; milling and polishing; milling and glazing). In addition, the authors note that glazing reduces the fracture strength of zirconia. Furthermore, while our research has confirmed the impact of the ratio between two crystalline phases (t-ZrO_2_ vs. m-ZrO_2_) on the mechanical properties of samples, it is noteworthy that this ratio also significantly affects translucency due to the distinct refractive indices of these phases [43,44].

As previously mentioned, it is important to note that there are additional surface treatment methods for zirconia that offer a wide range of benefits. For example, photo-functionalization of zirconia surfaces using ultraviolet UVC and UVB light has been shown to induce beneficial effects on fibroblasts [24,26] as well as on the peri-implant epithelial attachment zone [25]. Marques et al. (2023) [27] reported that laser-treated zirconia implant surfaces (Nd:YAG laser) induce a favorable biological response from osteoblasts. Furthermore, a study by Yang et al. (2021) [29] confirmed that cold atmospheric plasma treatment effectively destroys Streptococcus mutans from zirconia surfaces. Plasma treatment also appears to enhance the adhesion of gingival epithelial cells to zirconia surfaces [30]. In addition, a TiO_2_ coating of zirconia implant abutments has been shown to prevent bacterial colonization [31] and promote rapid and strong cellular attachment [33]. A recent study by Hu et al. (2023) [35] explored the hydrothermal synthesis of zinc oxide (ZnO) nanocrystals on zirconia abutment surfaces; the findings suggest that this method facilitates the formation of an early biological seal between the implant abutment and surrounding soft tissue, contributing positively to the long-term stability of the implant.

Our study highlighted the fact that sandblasting induces the formation of monoclinic zirconia, and with the help of hydrothermal treatment of zirconia samples at 134 °C/2–3 bar, it is possible to identify which surface treatments can determine the transformation of the tetragonal form into the monoclinic form. Also, as mentioned before, the polished zirconia samples had the best structural stability, and sandblasting with particles that exceed 30 μm in diameter should be avoided, especially on translucent zirconia.

The results of our study have clinical and practical relevance, as they contribute to a clearer understanding of how various surface treatments influence the phase composition and microstructure of zirconia, factors that ultimately impact the long-term in vivo performance of such restorations. Since zirconia restorations undergo surface treatments after fabrication to enable their adhesive cementation to the remaining dental hard tissues or, in the case of implant-supported restorations, to metal components [8,9], it is essential that these treatments do not compromise the material properties [45,46,47,48]. In this context, there is an ongoing interest in eliminating sandblasting as a standard step in the surface preparation of zirconia for adhesive cementation. Alternatives (such as hot acid etching using a cocktail solution) are being explored to enhance the specific surface area for bonding without adversely affecting the integrity of the zirconia (D’Alessandro et al.) [49].

The limitations of our study could include the relatively small number of samples tested and the selection of only two zirconia materials. Future research could expand upon this study by increasing both the number and variety of surface treatments applied to zirconia, conducting a broader range of experimental tests, and extending investigations into the clinical area.

## 5. Conclusions

Based on the in vitro evaluation of the influence of clinically common surface treatments on zirconia ceramics used for dental restorations, the following conclusions were drawn.

Sandblasting the surfaces of zirconia samples induced the formation of the monoclinic phase, with greater transformation observed when larger alumina particles were used.

Hydrothermal treatment at 134 °C/2–3 bar favored the transformation of tetragonal to monoclinic zirconia across all tested materials.

Sandblasted translucent monolithic zirconia exhibited pronounced degradation even after the first hydrothermal cycle, allowing for the detachment of monoclinic particles from the material surface. The surface defects induced by sandblasting—such as cracks and craters—likely contributed to the t-ZrO_2_ to m-ZrO_2_ transformation.

Zirconia samples dedicated to prosthetic restoration infrastructures demonstrated the highest structural stability among all groups.

Polished zirconia samples showed superior structural stability in both types of zirconia evaluated.

Manual polishing provided the smoothest surface quality among all tested surface treatment methods.

## Figures and Tables

**Table 1:** Details of the materials tested.

Materials Tested in the Study: Trade Name	Manufacturer	Material Type	Additional Material Details Tested in the Study
CORiTECH Zr	IMES-ICORE GmbH, Eiterfeld, Hessen, Germany	Zirconia (ZrO_2_) dedicated to dental prosthetic restoration infrastructures 3Y-TZP *	SKU: 5250079814 LOT: 4031642012 PRODUCED: 17 October 2016 EXPIRATION: 16 October 2026 DISC DIMENSIONS: 98 × 14 mm
CORiTECH Zr transpa	IMES-ICORE GmbH, Eiterfeld, Hessen, Germany	Super translucent multilayered monolithic zirconia 3Y-TZP *	SKU: 5250109814 LOT: 5031806004 PRODUCT: 4 February 2018 EXPIRATION: 4 February 2028 DISC DIMENSIONS: 98.5 × 14 mm

* 3 mol% yttria-stabilized tetragonal zirconia polycrystal.

**Table 2:** Details of the zirconia samples included in the study, their final codification, and their number.

Material	CORiTECH Zr S1 (24 Samples)		CORiTECH Zr Transpa S2 (24 Samples)	
1st sub-group Pre-sintered zirconia	S1-0 4 samples		S2-0 4 samples	
2nd sub-group Sintered zirconia	S1-1 4 samples		S2-1 4 samples	
3rd sub-group Sintered and sandblasted zirconia	S1-2a 4 samples 30 μm Al_2_O_3_	S1-2b 4 samples 50 μm Al_2_O_3_	S2-2a 4 samples 30 μm Al_2_O_3_	S2-2b 4 samples 50 μm Al_2_O_3_
4th sub-group Sintered and polished zirconia	S1-3 4 samples		S2-3 4 samples	
5th sub-group Sintered, polished, and glazed zirconia	S1-4 4 samples		S2-4 4 samples	

**Table 3:** Presentation, in summary, of the interventions performed on the zirconia samples used in the study.

Intervention	Sintering	Sandblasting 30 μm	Sandblasting 50 μm	Pre-Polishing and Polishing	Glazing
S1-1 and S2-1	✓				
S1-2a and S2-2a	✓	✓			
S1-2b and S2-2b	✓		✓		
S1-3 and S2-3	✓			✓	
S1-4 and S2-4	✓			✓	✓

## Data Availability

The original contributions presented in the study are included in the article; further inquiries can be directed to the corresponding author/s.

## References

[1] Silva L.H.D., Lima E.D., Miranda R.B.D.P., Favero S.S., Lohbauer U., Cesar P.F. (2017). Dental ceramics: A review of new materials and processing methods. Braz. Oral Res..

[2] Alqutaibi A.Y., Ghulam O., Krsoum M., Binmahmoud S., Taher H., Elmalky W., Zafar M.S. (2022). Revolution of Current Dental Zirconia: A Comprehensive Review. Molecules.

[3] Kim H.-K. (2020). Optical and Mechanical Properties of Highly Translucent Dental Zirconia. Materials.

[4] Huang B., Chen M., Wang J., Zhang X. (2024). Advances in zirconia-based dental materials: Properties, classification, applications, and future prospects. J. Dent..

[5] Kongkiatkamon S., Rokaya D., Kengtanyakich S., Peampring C. (2023). Current classification of zirconia in dentistry: An updated review. PeerJ.

[6] (2015). Implants for Surgery—Ceramic Materials Based on Yttria-Stabilized Tetragonal Zirconia (Y-TZP).

[7] Cho Y.-E., Lim Y.-J., Han J.-S., Yeo I.-S.L., Yoon H.-I. (2020). Effect of Yttria Content on the Translucency and Masking Ability of Yttria-Stabilized Tetragonal Zirconia Polycrystal. Materials.

[8] Sailer I., Gottner J., Känel S., Hämmerle C.H.F. (2009). Randomized controlled clinical trial of zirconia-ceramic and metal-ceramic posterior fixed dental prostheses: A 3-year follow-up. Int. J. Prosthodont..

[9] Blatz M.B., Vonderheide M., Conejo J. (2018). The Effect of Resin Bonding on Long-Term Success of High-Strength Ceramics. J. Dent. Res..

[10] Guilardi L.F., Pereira G.K.R., Giordani J.C., Kleverlaan C.J., Valandro L.F., Rippe M.P. (2019). Effect of zirconia surface treatment, resin cement and aging on the load-bearing capacity under fatigue of thin simplified full-contour Y-TZP restorations. J. Mech. Behav. Biomed. Mater..

[11] Chevalier J., Gremillard L., Virkar A.V., Clarke D.R. (2009). The Tetragonal-Monoclinic Transformation in Zirconia: Lessons Learned and Future Trends. J. Am. Ceram. Soc..

[12] Pandoleon P., Kontonasaki E., Kantiranis N., Pliatsikas N., Patsalas P., Papadopoulou L., Zorba T., Paraskevopoulos K., Koidis P. (2017). Aging of 3Y-TZP dental zirconia and yttrium depletion. Dent. Mater..

[13] Elzoughary A.A., Hamza T.A.R., Metwally M.F. (2024). Effect of hydrothermal aging on color stability and translucency of two zirconia generations compared to lithium disilicate ceramics. J. Dent. Res. Dent. Clin. Dent. Prospect..

[14] Bergamo E.T., Campos T.M., Lopes A.C., Cardoso K.B., Gouvea M.V., de Araújo-Júnior E.N., Witek L., Gierthmühlen P.C., Coelho P.G., Jalkh E.B.B. (2021). Hydrothermal aging affects the three-dimensional fit and fatigue lifetime of zirconia abutments. J. Mech. Behav. Biomed. Mater..

[15] https://www.krbec.cz/uws_files/ke_stazeni/imes_icore_dental_catalogue_en_2018.pdf.

[16] http://colombschell.fr/wp-content/uploads/2015/07/SDB_CORiTEC-Zr-transpa_lmil-chh-ch_Rev_1.2-eng.pdf.

[17] https://www.ivoclar.com/en_li/products/equipment/programat-sinter-furnace-s2.

[18] Davidowitz G., Kotick P.G. (2011). The Use of CAD/CAM in Dentistry. Dent. Clin. North Am..

[19] https://www.ivoclar.com/ro_ro/products/accessories/optragloss.

[20] Linkevicius T., Valantiejiene V., Alkimavicius J., Gineviciute E., Andrijauskas R., Linkeviciene L. (2020). The Effect of a Polishing Protocol on the Surface Roughness of Zirconium Oxide. Int. J. Prosthodont..

[21] Fischer N.G., Wong J., Baruth A., Cerutis D.R. (2017). Effect of Clinically Relevant CAD/CAM Zirconia Polishing on Gingival Fibroblast Proliferation and Focal Adhesions. Materials.

[22] Karthigeyan S., Ravindran A.J., Bhat T.R.R., Nageshwarao M.N., Murugesan S.V., Angamuthu V. (2019). Surface modification techniques for zirconia-based bioceramics: A review. J. Pharm. Bioallied Sci..

[23] Silva G.C. (2020). A straightforward technique to obtain a subgingival nonglazed polished zirconia area in monolithic implant-supported prostheses. J. Prosthet. Dent..

[24] Roy M., Corti A., Daniele S., Martini C., Cavallini C., Piosik A., Pompella A., Roy R.A. (2021). Early changes of ECM-related gene expression in fibroblasts cultured on TiO_2_, ZrO_2_ and PEEK: The beneficial effects of UVC photofunctionalization. J. Photochem. Photobiol..

[25] Razali M., Ngeow W.C., Omar R.A., Chai W.L. (2021). An In-Vitro Analysis of Peri-Implant Mucosal Seal Following Photofunctionalization of Zirconia Abutment Materials. Biomedicines.

[26] Wen Y., Dong H., Lin J., Zhuang X., Xian R., Li P., Li S. (2023). Response of Human Gingival Fibroblasts and Porphyromonas gingivalis to UVC-Activated Titanium Surfaces. J. Funct. Biomater..

[27] Marques A.F.S., Loureiro F.A.P., Sahoo N., Marques J.R.O.F., da Cruz M.F.B., Mata A.D.S.P.D., Caramês J., Silva F.S.C.P.D., Carvalho Ó.S.N. (2023). Nd-YAG Laser Texturing of Zirconia Implant Surfaces. Lasers Manuf. Mater. Process..

[28] Esfahanizadeh N., Motalebi S., Daneshparvar N., Akhoundi N., Bonakdar S. (2016). Morphology, proliferation, and gene expression of gingival fibroblasts on Laser-Lok, titanium, and zirconia surfaces. Lasers Med. Sci..

[29] Yang Y., Zheng M., Jia Y.-N., Li J., Li H.-P., Tan J.-G. (2021). Time-dependent reactive oxygen species inhibit Streptococcus mutans growth on zirconia after a helium cold atmospheric plasma treatment. Mater. Sci. Eng. C.

[30] Gineviciute E., Alkimavicius J., Andrijauskas R., Sakalauskas D., Linkeviciene L., Tomas L., Pros D. (2021). Comparison of different cleaning procedures to decontaminate zirconium oxide surface after polishing. Int. J. Prosthodont..

[31] Zühlke A., Gasik M., Shahramian K., Närhi T., Bilotsky Y., Kangasniemi I. (2021). Enhancement of Gingival Tissue Adherence of Zirconia Implant Posts: In Vitro Study. Materials.

[32] Areid N., Riivari S., Abushahba F., Shahramian K., Närhi T. (2023). Influence of Surface Characteristics of TiO_2_ Coatings on the Response of Gingival Cells: A Systematic Review of In Vitro Studies. Materials.

[33] Wang C., Wang X., Lu R., Gao S., Ling Y., Chen S. (2021). Responses of human gingival fibroblasts to superhydrophilic hydrogenated titanium dioxide nanotubes. Colloids Surf. B Biointerfaces.

[34] Mohammed D.H., Jassim R.K. (2023). Optimizing the Surface Properties of Zirconium Implants with Germanium Coating. J. Biomim. Biomater. Biomed. Eng..

[35] Hu J., Atsuta I., Luo Y., Wang X., Jiang Q. (2023). Promotional Effect and Molecular Mechanism of Synthesized Zinc Oxide Nanocrystal on Zirconia Abutment Surface for Soft Tissue Sealing. J. Dent. Res..

[36] Maruo Y., Yoshihara K., Irie M., Nishigawa G., Nagaoka N., Matsumoto T., Minagi S. (2020). Flexural properties, bond ability, and crystallographic phase of highly translucent multi-layered zirconia. J. Appl. Biomater. Funct. Mater..

[37] Hammoudeh H., Carracho L., Beard C., Razzoog M. (2024). Effect of different surface and heat treatments on the surface roughness, crystallography, and phase composition of high translucency zirconia for monolithic restorations. J. Prosthet. Dent..

[38] Jamali M., Ezoji F., Esmaeili B., Khafri S. (2024). Comparative effects of glazing versus polishing on mechanical, optical, and surface properties of zirconia ceramics with different translucencies. Clin. Exp. Dent. Res..

[39] Ozturk I., Caglar I., Duymus Z.Y. (2022). The effect of adjustment and finishing procedure on roughness, strength, and phase transformation of monolithic zirconia. Clin. Oral Investig..

[40] Sarabi N., Mohammadi-Bassir M., Fadavi F., Rezvani M.B., Dehestani-Ardakani F., Labbaf H. (2024). Effect of Hydrothermal, Chemical, and Mechanical Degradation on Flexural Strength and Phase Transformation of Ground, Glazed, and Polished Zirconia. Front. Dent..

[41] Hatanaka G.R., Polli G.S., Adabo G.L. (2020). The mechanical behavior of high-translucent monolithic zirconia after adjustment and finishing procedures and artificial aging. J. Prosthet. Dent..

[42] Vila-Nova T.E., de Carvalho I.H., Moura D.M., Batista A.U., Zhang Y., Paskocimas C.A., Bottino M.A., Souza R.O. (2020). Effect of finishing/polishing techniques and low temperature degradation on the surface topography, phase transformation and flexural strength of ultra-translucent ZrO_2_ ceramic. Dent. Mater..

[43] Dash A., Kim B.-N., Klimke J., Vleugels J. (2019). Transparent tetragonal-cubic zirconia composite ceramics densified by spark plasma sintering and hot isostatic pressing. J. Eur. Ceram. Soc..

[44] Xu P., Wang H., Cui W., Chen Q., Tu B., Sang X., Wang W., Fu Z. (2022). ZnO·2.7 Al_2_O_3_ nanocomposite with high optical transparency. J. Am. Ceram. Soc..

[45] Özarslan M., Can D.B., Avcioglu N.H., Çalışkan S. (2022). Effect of different polishing techniques on surface properties and bacterial adhesion on resin-ceramic CAD/CAM materials. Clin. Oral Investig..

[46] Go H., Park H., Lee J., Seo H., Lee S. (2019). Effect of various polishing burs on surface roughness and bacterial adhesion in pediatric zirconia crowns. Dent. Mater. J..

[47] Zeng J., Song J., Zhang Y., Yang Z., Nie E., Zhang C., Jiang R. (2023). Surface roughness and bacteria adhesion of full zirconia restoration after different polishing treatment. Chin. J. Tissue Eng. Res..

[48] Toma F.R., Porojan S.D., Vasiliu R.D., Porojan L. (2023). The Effect of Polishing, Glazing, and Aging on Optical Characteristics of Multi-Layered Dental Zirconia with Different Degrees of Translucency. J. Funct. Biomater..

[49] D’ALessandro C., Josic U., Mazzitelli C., Maravic T., Graham L., Barausse C., Mazzoni A., Breschi L., Blatz M.B. (2024). Is zirconia surface etching a viable alternative to airborne particle abrasion? A systematic review and meta-analysis of in vitro studies. J. Dent..

